# Natural Killer cells at the frontline in the fight against cancer

**DOI:** 10.1038/s41419-024-06976-0

**Published:** 2024-08-23

**Authors:** Loïs Coënon, Mannon Geindreau, François Ghiringhelli, Martin Villalba, Mélanie Bruchard

**Affiliations:** 1 IRMB, Univ Montpellier, INSERM, CHU Montpellier, Montpellier, France; 2 Equipe TIRECs, Labellisée Ligue Contre le Cancer, Centre de Recherche INSERM CTM-UMR1231, Dijon, France; 3 University of Bourgogne Franche-Comté, Dijon, France; 4 Platform of Transfer in Biological Oncology, Georges-François Leclerc Cancer Center, Dijon, France; 5 Institut du Cancer Avignon-Provence Sainte Catherine, Avignon, France; 6 IRMB, Univ Montpellier, INSERM, CHU Montpellier, CNRS, Montpellier, France

**Keywords:** Innate lymphoid cells, Immunosurveillance, Cell death and immune response

## Abstract

Natural Killer (NK) cells are innate immune cells that play a pivotal role as first line defenders in the anti-tumor response. To prevent tumor development, NK cells are searching for abnormal cells within the body and appear to be key players in immunosurveillance. Upon recognition of abnormal cells, NK cells will become activated to destroy them. In order to fulfill their anti-tumoral function, they rely on the secretion of lytic granules, expression of death receptors and production of cytokines. Additionally, NK cells interact with other cells in the tumor microenvironment. In this review, we will first focus on NK cells’ activation and cytotoxicity mechanisms as well as NK cells behavior during serial killing. Lastly, we will review NK cells’ crosstalk with the other immune cells present in the tumor microenvironment.

## Facts

- NK cells are first line defenders in cancer, by natural cytotoxicity and by mediating ADCC making them crucial components for cancer immunosurveillance.
- NK cells have at their disposal several weapons in their arsenal such as cytotoxic molecules, cytokines, or death ligands, to destroy cancer cells.
- Interaction between NK cells and innate or adaptative immune cells in the tumor microenvironment are various and complex, contributing to shape the anti-tumor response.

## Open questions

- How fundamental knowledge learnt in pre-clinical studies can help improving NK cell-based therapy?
- To which extent ex vivo expanded NK cells used in therapy interact with immune cells in the tumor microenvironment?
- There are multiple NK cell subsets, but their specific in fighting cancer are unknown.

## Introduction

Natural Killer (NK) cells were first described in mice by Rolf Kiessling, Eva Klein and Hans Wigzell in the 1970s as “naturally occurring killer lymphocytes” able to destroy murine leukemia cells [1, 2]. Since then, NK cells have been thoroughly studied and relatively well described as part of the innate lymphoid cell (ILC) family.

NK cells are first-line defenders of the immune response against infectious diseases [3] and cancer [4] but they are also important in autoimmune [5, 6] and metabolic diseases [7]. They provide rapid responses because they do not require prior priming. They recognize target cells using germline-encoded surface receptors able to recognize activating and inhibitory signals. If more activating receptors are engaged, NK cells engage in two different pathways: directly kill the target cell or shape the tumor microenvironment through cytokine production and direct cell-cell interactions.

NK cells are defined as CD56^+^; CD3^-^ and can be overall classified into two distinct subsets: CD56^bright^ CD16^-^ and CD56^dim^ CD16^+^. Several reports suggested that CD56^bright^ NK cells can differentiate into CD56^dim^ subset [8, 9]. The first subset is thought to be a not fully mature group of NK cells which account for 5-10% of peripheral blood NK cells and can secrete large amounts of cytokines [10]. The second one is mature, produces lower cytokines and exerts strong antibody-dependent and independent cytotoxicity [11]. Moreover, less mature CD56^dim^ NK cells can strongly respond to several cytokines including IL-12, IL-15 and IL-18, whereas more mature CD56^dim^ NK cells lose this ability but increase their cytotoxic function [12]. Despite the above-mentioned classification, stating that CD56^bright^ NK cells are mainly cytokines producers, it is worth mentioning that CD56^bright^ NK cells can exert strong cytotoxic response in vitro and in vivo against tumor cells after IL-15 priming [13].

Two models exist regarding NK cell development in tissues. First, NK cells develop within bone marrow or secondary lymphoid organs. CD56^bright^ and CD56^dim^ NK cells migrate from secondary lymphoid organs or bone marrow respectively to peripheral organs, in which tissue-specific differentiation occurs. The second model proposes that NK cells migrate from bone marrow in an early development state to tissues, where they mature. This model is based on the fact that NK cell precursors have been found in several peripheral tissues such as the guts, uterus and liver [12]. Nevertheless, it is established that the compartment in which NK cells migrate drives their maturation to grant them tissue-specific phenotype [14, 15].

NK cells form a very diversified cell population that are found in most tissues. Indeed, NK cells reside in high numbers in the peripheral blood, in the lungs, uterus, liver and more rarely in lymph nodes and tonsils. For example, NK cells can represent up to 50% of the lymphocytes in the uterus [12]. Moreover, CD56^bright^ NK cells are more frequent in the guts, tonsils, lymph nodes and the skin, whereas in the liver, the lungs and the spleen NK cells are mainly CD56^dim^ [12].

T-bet and Eomes have long been reported as major drivers of NK cells development in mice [16]. In humans, T-bet and Eomes knock-out in NK cells reduce IFNγ, perforin (Prf) and granzyme B (GrzB) production as well as proliferation, thus impairing NK cells anti-tumor function in tumor-bearing NSG mice model [17]. Another transcription factor, IRF2, has recently been implicated in human NK cell proliferation and functional maturation [18]. Thus, NK cell’s development relies on several transcription factors, conferring them potent cytotoxic function.

In this review, we will focus on NK cell’s role in fighting cancer, reviewing knowledge from both human and mice studies. A summary of the difference between human and mice NK cell has been published [19]. First, we will focus on NK cells’ cytotoxic arsenal and then, on NK cell’s communication with other immune cells in the tumor microenvironment (TME).

## NK cell cytotoxic Arsenal

### Immunosurveillance and importance in cancer

NK cells are known to be one of the main drivers of cancer immunosurveillance. NK cells are patrolling within the body hunting for abnormal cells, such as transformed cells, preventing tumor development [20]. Moreover, a link between the cytotoxic capability of peripheral blood lymphocytes and cancer occurrence in a Japanese cohort has been unveiled [21]. It is most likely that NK cells account for a significant part of the cytotoxic PBMCs, supporting their importance in immunosurveillance [21]. In this line, NK cells anti-tumor activity is important for tumor control. Indeed, NK cell exhaustion and cytokine secretion defect have been linked to impaired survival in patients with newly diagnosed multiple myeloma [22], and low NK cell count in the peripheral blood is associated with impaired survival in follicular lymphoma patients [23]. Additionally, tumor-infiltrating NK cells are linked to better survival in several solid cancers including head and neck cancer, breast cancer, lung cancer and others [24]. NK cells are also known to target circulating tumor cells [25] that underwent epithelial to mesenchymal transition, which is part of the metastatic process [26]. Indeed, circulating tumor cells express ligands such as E-cadherin and CDMA1 [27], which are recognized by NK cells and induce their cytotoxicity. Furthermore, in a mouse model, NK cell depletion led to an increase in the number of pulmonary metastases whereas T cell depletion did not impact the number but the area of the metastatic foci. This paper highlights the ability of NK cells to “clear” circulating cancer cells and their role in immunosurveillance [28]. Although numerous works place NK cell as one of the main protagonists in the fight against cancer, it is important to balance this by emphasizing that NK cell’s functions are greatly impaired in the TME. Immunosuppressive cells, such as M2 macrophages or regulatory T cells, soluble factors, such as IL-10 or TGF-β, and membrane-bound ligands, such as immune checkpoint inhibitors including PD-1 and TIM-3, are all factors able to inhibit NK cells activity [29]. Cell-cell interaction modulating NK cell activity will be specifically discussed later in this review. Moreover, it was recently shown in a mice model that NK cell loss of effector functions happens rapidly after NK cell entry in the tumor, revealing a probable short-term action of NK cells after tumor engagement [30].

### Immune synapse formation: stick close to your enemy

NK cells’ cytotoxic activity is mainly mediated by the release of lytic granules which contain cytotoxic molecules. This tightly regulated mechanism occurs through sequential steps [31]. NK cells interact with target cells through several receptors. Some of them are activating, others inhibitory. If activating signals are superior to inhibitory, the immune synapse (IS) is formed. The IS formation is initialized by integrins ligation expressed by NK cells such as LFA-1 to its cognate ligands ICAM-1/2 [32–34]. This interaction will stabilize the IS thanks to a firm adhesion to the target cell [35] and also induce an actin reorganization within the NK cells through clustering of activating receptors. Interestingly, CD56, the major surface recognition marker of NK cells also plays an adhesion role [36]. More recently, CD56 engagement with a target cell was showed to be necessary for IS formation, intracellular target phosphorylation, and degranulation, as CD56 KO NK cell line failed to do so [37]. Upon engagement with ICAM-1/2, LFA-1 allows lytic granule guidance to the IS and in addition induces strong degranulation through mechanosensing [38]. Intracellular signals from clustered activating receptors induce MTOC polarization which drives scattered lytic granules within NK cells cytosol to converge to the IS by allowing them to travel on microtubules [39]. Once near the IS, lytic granules will then move onto the actin filaments until reaching the cell membrane where their content will be locally released in the synaptic cleft onto the target cell through exocytosis [40]. Finally, IS maintenance was shown to be an active process, since sustained signaling from engaged activating receptors maintains it during the killing event [41].

### NK cells activating receptors

NK cell cytotoxicity is governed by a plethora of activating and inhibitory receptors expressed at their membrane. By interacting with target cell ligands, a balance between activating and inhibiting signals will be integrated within the NK cell and decide the fate of the target cell: live or die [42].

Activating receptors are associated with different intracellular chains that transduce signals. For instance, Natural Cytotoxic Receptors (NCR), NKG2C, an activating receptor belonging to the NKG2 family, and CD16a interact with immunoreceptor tyrosine-based activation motif (ITAM)-containing domains such as DAP12 for the NCR and NKG2C, or CD3ζ and FcεRIγ for CD16a [43]. In the case of NKG2D, another receptor of the NKG2 family, it is associated with DAP10 [44]. In any case, when these activating receptors bind to their ligand, the intracellular chains will be phosphorylated by protein kinase from the Src family. This event will trigger various intracellular pathways including Ca^2+^ flux, ultimately leading to exocytosis and gene transcription [44]. On the other hand, inhibitory receptors signal through immunoreceptor tyrosine-based inhibitory motif (ITIM) domains. ITIM domains recruit phosphatases that dephosphorylate several targets, inhibiting NK cell function [44]. In this section, we will focus only on the activating receptors, responsible for NK cell antitumor functions.

### Natural cytotoxic receptors

The members of the NCR family, namely NKp30, NKp44 and NKp46, are almost all ITAM-bearing receptors and thus activating receptors, except for some variants. In humans, six NKp30 variants have been described, some being activating and others potentially inhibitory [43]. Nonetheless, NKp30 was reported to induce NK cell activation and cytotoxicity upon binding to its ligands BAT3 [45] and B7-H6 [46]. Both antigens have been associated with poor outcomes in patients suffering from gastrointestinal cancer [47], and NK cells’ low NKp30 surface expression is linked to poor patients’ outcomes in hematological and solid cancer [48–51].

NKp44 can bind to PCNA, PDGF-DD and NID1 [43], 21spe-MLL5 and cell-surface heparan sulfate proteoglycans (HSPG) [52]. There are 3 different isoforms of NKp44: NKp44-1, -2 and -3. NKp44-1 possesses an ITIM domain in its cytoplasmic tail, while the two others have an ITAM domain. NKp44-1 binding to PCNA decreases NK cell activation and its expression is associated with low survival in acute myeloid leukemia patients [53]. In line with this, in vitro PCNA blockade using monoclonal antibodies (mAbs) enhances NK cells mediated tumor cell killing [54]. On the other hand, NKp44/PDGF-DD interaction induces cytokine production by NK cells. Moreover, NKp44 high expression is linked to increased survival in low grade glioma patients [55]. NKp44 binding to NID1, a component of the extracellular matrix, inhibits PDGF-DD-induced cytokines production [56]. Accordingly, NID1 expression is linked to poor patient outcomes in low-grade gliomas [57]. MLL5 is primarily a nuclear protein but its isoform, 21spe-MLL5, is found in the cytoplasm and at the cell surface of cancer cells. Its recognition by NKp44 trigger NK cell’s cytotoxicity [58]. Finally, NKp44 can recognize cell surface HSPG, whose expression vary between cell type and can be modified in tumor cells [59]. Although HSPG recognition by NKp44 triggers IFNγ secretion [60], other report tends to show that activation of a cytotoxic activity is most likely not happening [61].

NKp46 is involved in the killing of various tumor cells since NKp46 blockade using mAbs drastically decreased NK cell’s ability to destroy tumor cells [62]. Moreover, in several cancer types, low NKp46 transcription and surface expression on NK cells have been associated with poor patient outcomes [23, 63, 64]. In colorectal cancer patients, CD56^dim^ NK cells express less NKp46 than in healthy donors, suggesting an anti-tumor role for NKp46 [65]. Nevertheless, the NKp46 ligand externalized calreticulin (ecto-CRT) was only recently described on tumor cell surface [66]. This protein is normally found in the endoplasmic reticulum (ER) where it acts as a chaperone. However, during ER stress induced by immunogenic cell death inducers, ecto-CRT is translocated to the plasma membrane [67]. Hence, chemotherapy-treated tumor cells express ecto-CRT at their membrane, becoming more sensitive to NK cell activity through, at least in part, ecto-CRT recognition by NKp46.

### NKG2 family

Another main NK cell activating receptor is NKG2D, which binds to several ligands, some of them being referred to as “stress ligands” such as MHC class I chain-related proteins A and B (MICA/B) and the UL16 binding protein (ULBP) molecules family [68]. Intracellular signaling of NKG2D is mediated by DAP10, a non-bearing ITAM domain intracellular chain [44]. The importance of this receptor for tumor immunosurveillance was demonstrated using a murine model of NKG2D deficiency in which tumor incidence was highly increased [69]. In fact, NKG2D ligands have been found at the surface or tumor cells or in soluble form in a wide range of cancers, especially MICA [70]. More recently, NKG2D’s role in clearing circulating tumor cells was highlighted in a study showing that recognition of its cognate ligands is potentiated in fluid shear stress through mechanosensing [71]. In the clinic, a high level of NKG2D ligands’ expression at the tumor cells membrane is correlated with better prognosis and increased NK cell infiltration in the tumor [72]. Interestingly, a recent report demonstrated that CD8 + T cells are able to eliminate MHC-I negative tumor cells in mice model and in vitro with human cells through the NKG2D axis [73]. Thus, NKG2D-mediated tumor clearance could also be, at least in part, due to CD8 + T cells activity.

NKG2C is an activating receptor that works in heterodimers with CD94 [74]. CD94/NKG2C recognizes HLA-E which is expressed by several cell types including tumor cells [75]. CD94/NKG2C signaling is driven by an ITAM-bearing chain, DAP12 [74]. The amount of NKG2C-positive NK cells appears to be beneficial in hematopoietic cell transplant (HCT). First, they were associated with a better response after HCT in patients with hematological malignancies [76]. Next, NK cells are known to be involved in silencing graft-versus-host-disease (GvHD) mediated by the donor T cells by destroying them [77]. More specifically, the NKG2C-positive NK cells seem to be critical in this context, as patients with severe GvHD were found to have lower percentage of this NK cell subset [78]. Another potential ligand for CD94/NKG2C is membrane Heat shock protein 70 (Hsp70). This stress protein is expressed at an elevated level at the surface of several types of solid cancer cells [79] and was shown to sensitize them to NK cells activity [80]. In this context, CD94 was found to be involved in the recognition of Hsp70 [81].

### DNAM1

DNAM1/CD226 is a potent activating receptor that has been shown to recognize two ligands overexpressed by tumor cells [82], namely PVR/CD155 which is considered as the main ligand [83] and Nectin2/CD112 [84]. CD155 expression correlates with bad prognosis in several solid cancers, pointing out the importance of this ligand for tumor control [85, 86]. Consistently, DNAM1 deficient mice show accelerated tumor growth compared to WT mice [87], although not only NK cells but also CD8^+^ T cells express this receptor [88]. Hence, the observed effect might be the sum of impede function of both NK cells and CD8^+^ T cells.

### CD16a/FcγRIIIa

Last, but not least, the strongest degranulation inducer is probably the CD16a receptor, which belongs to the Fc-gamma receptor family, which includes receptors for the Fc moiety of IgG [89]. CD16a is a transmembrane receptor that possesses two extracellular immunoglobulin domains [90]. CD16a short cytoplasmic tail does not contain any signaling component; hence non-covalently interacting ITAM-bearing intracellular chains are needed to induce strong intracellular signals [91]. Hence, antibody-coated cells can be recognized by CD16a expressing NK cells, leading to NK cell degranulation. Of interest, a functional polymorphism has been described, the F158V, the V variant being the one with the best affinity for the Fc moiety of IgG [92].

### Lytic granules: NK cells’ nuclear bomb

As described above, NK cells as well as other cytotoxic T lymphocytes carry lytic granules in their cytosol. These granules contain several cytotoxic molecules, such as Prf, GrzB and granulysin (GNLY). Interestingly, NK cells constitutively express these granules, whereas cytotoxic T lymphocytes require activation [93]. This notably could help to explain the rapid functions of NK cells in contrast to CD8^+^ T cells.

Recently, an article reviewed the steps leading to the biogenesis of GrzB [40]. Briefly, GrzB is produced as a zymogen composed of the catalytic part, a peptide signal and an inhibitory peptide. Once it arrives in the ER, it translocates to the Golgi apparatus and then into lytic granules. Inside lytic granules, the inhibitory dipeptide is cleaved, however, GrzB activity is still inhibited through interaction with a serglycin molecule. Concerning Prf, it is produced in the ER in an inactive state, and it translocates to the Golgi apparatus then in lytic granules [40]. The exact mechanism remains unclear thus further research is needed [40].

Upon release in the IS, Prf will form a pore into the target cell’s membrane allowing GrzB to penetrate the cytoplasm [94, 95]. GrzB will then initiate apoptosis by cleaving several substrates such as Bid or caspase-3 [96–98]. Of note, it is known that some target cells can be destroyed through necrosis, pointing out an osmotic lysis driven by Prf only [99]. Additionally, GNLY also displays pore-forming activities, allowing not only GrzB to enter target cells [100] but also directly trigger target cell’s apoptosis by inducing cytochrome C release from mitochondria [101].

Recently, it has been described that Prf and GrzB can be released in complex with thrombospondin-1 (TSP-1) from lytic granules, forming structures termed SMAPs standing for supramolecular attack particles. SMAPs may contribute to the efficiency of the delivery of GrzB and Prf to the target cell. Interestingly, SMAPs size from NK cells were found to be larger than those from T cells [102].

NK cells are protected from the Prf/GrzB cytotoxic activity after release through the CD107a/LAMP-1 translocation at the membrane. Originally present at the surface of the lytic granule, CD107a will be expressed at the surface plasma membrane after exocytosis and thus prevent pore-formation [103].

Finally, target cells can confine GrzB in the endosomal compartment as a defensive measure. However, Prf is capable of permeabilizing endosomes thus releasing the sequestered GrzB into the cytosol [104].

### Delivering the kiss of death: TNF-related apoptosis-inducing ligands

Apart from lytic granules release, NK cells can rely on other weapons namely two TNF-related apoptosis-inducing ligands belonging to the TNF superfamily, TRAIL and FasL (CD95L). Upon binding to their cognate ligands, TRAIL R1–R2 and Fas receptor (CD95) respectively, apoptosis will occur inside the target cells [105, 106]. The apoptosis pathway triggered by death receptor engagement is the extrinsic pathway, contrary to cytotoxic molecules from lytic granules that trigger the intrinsic pathway. Fas and TRAIL ligands engagement induce a Death-Inducing Signaling Complex (DISC) formation mediating strong apoptotic intracellular signal through caspase 8 maturation and subsequently effector caspase 3, 6 and 7 activation [107].

FasL and TRAIL expression regulation are still a subject of research. FasL seems to be stored separately from Prf/GrzB in another type of lytic granule [108] and upon lytic granule-mediated target cell killing, is gradually expressed at the membrane [35, 109]. Regarding TRAIL, additional work is needed to decipher its intracellular localization and regulation in humans. Nevertheless, TRAIL surface expression seems to be IFNγ dependent, as demonstrated in IFNγ-deficient mouse models [110, 111]. Additionally, in a murine model, NKp46 has also been shown to regulate TRAIL membrane location. Indeed, NK cells from NKp46 deficient mice were devoid of membrane-bound TRAIL but were found intracellularly [112]. In humans NK cells, TRAIL surface expression is inducible through cytokines stimulation [113], similar to T cells on which TRAIL can only be found at the membrane after activation [114].

### IFNγ and TNF-α: the right and left arms of NK cells

NK cells are also well-known cytokine producers, in particular IFNγ and TNF-α. Cytokines are stored in different vesicles than lytic granules and are released upon NK cell’s activation [115]. However, the secretion pathway is different since lytic granules are secreted locally in the IS as mentioned above, whereas cytokines are widely released in the environment [116].

As pro-inflammatory cytokines, IFNγ and TNF-α promote other immune cell activation and can induce tumor cell apoptosis [117, 118]. TNF-α increases IFNγ production through signaling with its receptors TNFR2 [119], while IFNγ increased the motility and cytotoxicity of cytotoxic lymphocytes [120]. Taken together, these studies highlight the essential role of cytokines in mediating and supporting NK cell cytotoxicity, as well as communicating with other immune cells.

### Antibody-dependent-cell-cytotoxicity

Upon CD16a binding to IgG Fc, a potent cytotoxic mechanism known as Antibody-Dependent-Cell-Cytotoxicity (ADCC) is triggered, leading to target cell death. Although CD16a alone is enough to trigger ADCC, other receptors seem to positively modulate it as shown for LFA-1, CD38 or CD2 [121–123]. ADCC is one of the main mechanisms of action of some clinical mAbs. Indeed, patients harboring CD16 158 V NK cells show a better clinical response after rituximab (an antibody targeting CD20) treatment [124] and in trastuzumab (an antibody targeting HER2)-treated HER2^+^ breast cancer patients [125]. Moreover, it has been recently shown in mice models that high levels of intra-tumor hinge-cleaved IgG impaired NK cell function and tumor clearance, highlighting the importance of CD16a/IgG Fc interaction for NK cells anti-tumor activity [126]. In the clinic, tumor-infiltrating NK cells are predictive of pathological complete response in breast cancer patients who received anti-HER2 mAb [127]. In hematological cancer, peripheral blood NK cell count seems associated with follicular lymphoma and diffuse large B cell lymphoma (DLBCL) patients’ response to anti-CD20 mAb therapy [128]. Taken together, NK cell mediated ADCC appears to be an essential mechanism of several widely used clinical mAbs.

### Dynamics of NK cell’s cytotoxic activity

**Fig. 1:**
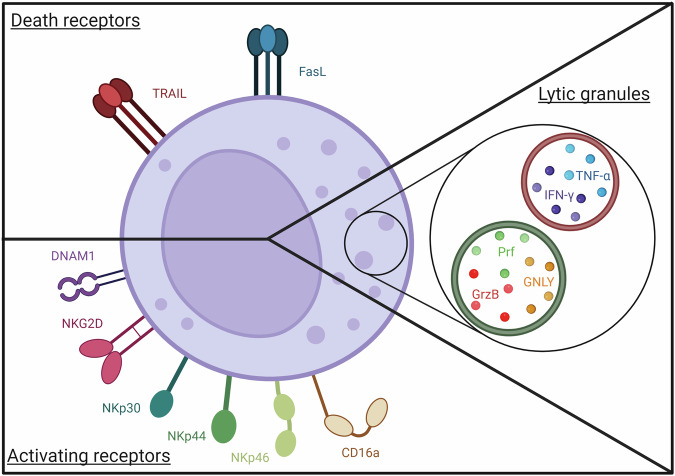
NK cell cytotoxic Arsenal. NK cells rely on activating receptors which lead to lytic granules content and cytokines release and on death receptors whose induce apoptosis. GNLY granulysin, GrzB granzyme B, Prf perforin. Created with BioRender.com.

NK cells rely on several killing pathways to achieve target cell elimination (Fig. 1). However, when, and how NK cells use which pathway is still an active field of study. After destroying a target cell, NK cells can find another target cell. This process is known as serial killing, in which NK cells destroy target cells one by one [129]. Primary NK cells carry about 60 lytic granules in their cytosol and they degranulate around 10% of their total granule per killing event [130]. This is consistent with previous findings showing that NK cells can kill up to 10 targets during serial killing [129]. Of note, 1% of NK cells’ lytic granules may be enough to kill a target cell, highlighting the exceptional potency of lytic granule content [130]. During serial killing, repeated stimulation of the CD16a pathway diminishes the amount of Prf secreted over time. Interestingly, switching the NK cells degranulation pathway to NKG2D restores the original amount of secreted Prf [131]. Thus, when NK cells use all available lytic granules, they switch their killing pathway toward death receptors-mediated cytotoxicity [113]. Indeed, FasL was shown to be expressed gradually at the membrane during serial killing concomitantly with CD107a expression, since it is stored in lytic granules. Interestingly, FasL-mediated target cell killing seems to account for only one, ultimate killing event [113]. Of note, death receptors-induced target cell apoptosis was shown to be a rather slow mechanism, while lytic granule-mediated cell death occurs faster [132].

IS termination and subsequent NK cell detachment from a target cell seem to be a critical step in serial killing. It was proposed that NK cells can interact and integrate signals from several targets at the same time. Indeed, it was shown that the first killing event is slower than the next ones, and that consecutive killing events are adjacent [91]. In line with this, others described that upon interacting with a new target cell, NK cells will detach faster from the previous one [41]. Moreover, the detachment process was found to be dependent on the successful killing of the target cell, and a failure was associated with an extensive cytokines release, including IFNγ [133]. Interestingly, a similar pattern can be found in studies reporting ADAM17 blockade strategies. ADAM17 is a metalloprotease responsible for the CD16a shedding after ADCC induction [134, 135]. Hence, blocking ADAM17 activity could increase NK cells mediated ADCC. In the end, it was shown that blocking ADAM17 using chemicals, mAbs or by generating NK cells bearing non-cleavable CD16a not only increased NK cells IFNγ secretion during ADCC assays [135, 136] but also reduced serial killing [131]. These findings are consistent with the above-mentioned study and point out the critical role of IS disassembly in NK cells serial killing [133].

### Analyzing NK cell interaction with tumor cells

The presence of NK cells that interact with tumor cells have recently been described in leukemia and lymphoma patients. NK cells interacting with tumor cells usually express the two CD45 isoforms CD45RA and CD45RO [137], and the interaction generates new NK cell subsets with different functionalities [138]. The anti-tumor NK cells perform trogocytosis on tumor markers facilitating their identification [137]. The following of this NK cell subset allows the surveying of the presence of tumor cells and the prognostic/theragnostic of the patient [139–141]. Moreover, trogocytosis can affect NK cell functionality [142–145] and, probably, the response to treatment [146].

## NK cells crosstalk with other immune cells: need help from some friends

In the TME, the NK cell lineage act as a veritable conductor of the anti-tumor immune response. NK cells cooperate through direct contact or soluble factor secretion with other immune cells and participate in their recruitment, maturation, polarization, differentiation and destruction. In turn, immune cells can have a positive impact on NK cells by increasing ADCC, IFNγ production, activating receptor expression, recruitment and cytotoxicity. However, some cells could also have a negative impact on NK cells by reducing their activation and cytotoxicity.

### NK cells cooperation with innate immune cells

#### Macrophages

Macrophages are phagocytic and antigen-presenting cells at the interface between innate and adaptive immunity. Tumor-associated macrophages (TAM) are divided into two subtypes: TAM1 with an anti-tumor activity and TAM2 with a pro-tumor activity. TAM1 are induced by IFNγ and LPS and produce cytokines such as TNF-α, IL-1β, IL-6, IL-12, nitric oxide synthase (iNOS) and reactive oxygen species (ROS). TAM2 are induced by cytokines such as IL-4, IL-10 and IL-13, and produce cytokines such as IL-10, TGF-β and VEGF [147, 148].

First, TAM1, by both soluble factor secretion and direct contact, increase CD69 expression (an activating marker), degranulation and IFNγ secretion of autologous resting NK cells [149]. The production of IFNγ by TAM induce the expression of IL-15Rα on NK cells and their IL-15 production. This cis-presentation increases the production of IFNγ by NK cells [149]. Interestingly, TAM1 also increase NK cell’s cytolytic efficiency by up-regulating NKG2D and NKp44 expression through IL-1β, IL-23 and IFN-β secretion [149]. Additionally, polyI:C-treated macrophages increase NKG2D expression on murine splenic CD49b^+^ NK cells and therefore, their cytotoxicity against several tumor cell lines. Interestingly, these murine macrophages express Qa-1 which is recognized by the inhibitory receptor NKG2A, thus avoiding to be killed by NK cells [150]. Monocyte-derived macrophages cultured with IFNλ/LPS produce IL-12 family cytokines which in turn induce human NK cells IFNγ production [151].

One study focused on the mechanism involved in NK cell-mediated tumor killing orchestrated by macrophages and dendritic cells (DCs). In particular, the C-type lectin receptor Dectin-1, which is expressed by macrophages and DCs, able recognition of N-glycan structures on tumor cells. This induces IRF5 activation and in turn downstream genes’ transcription. Overall, these events allow macrophages and DCs to activate NK cell’s anti-tumor activity against N-glycan-expressing tumor cells [152].

In contrast, TAM-derived TGF-β impairs NK cell function by decreasing their cytokine production, degranulation in humans [153, 154] and cytotoxicity in mice [155]. TAM also promote an exhausted phenotype CD27^low^ CD11b^high^ in mice [155]. In a mice model, Klose et al. demonstrated that targeting VEGF-A in myeloid cells increases chemotherapy delivery to the tumor bed and also increases NK cell recruitment after chemotherapy [156].

**Fig. 2:**
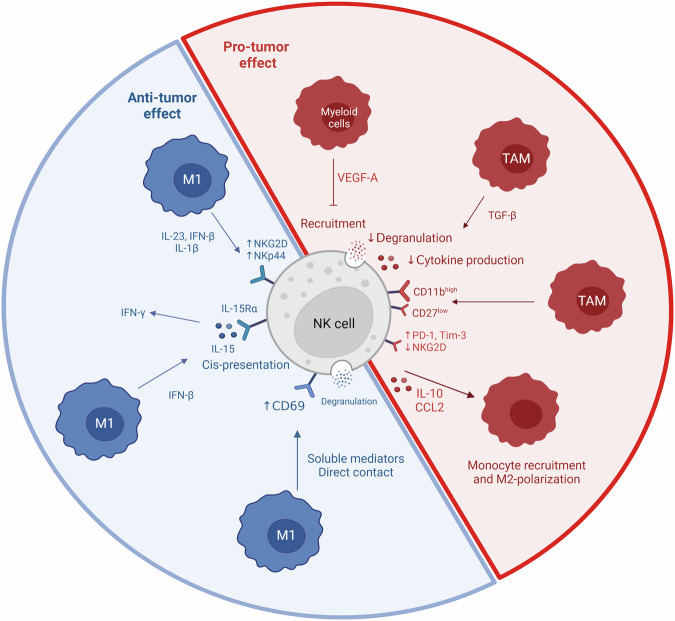
NK cells and macrophage interaction. Macrophages are innate immune cells divided into pro-tumor M1 macrophage and anti-tumor M2 macrophages. There are different interactions between NK cells and macrophages. Macrophages and notably M1 have an anti-tumor effect on NK cells: increase of activating receptors, cytokine production and degranulation. Macrophages also have a pro-tumor effect by inhibiting NK recruitment, degranulation, cytokine production and inducing an exhaustion phenotype. In turn, NK cells induce monocyte recruitment and M2 polarization. Created with BioRender.com.

NK cells can also negatively affect anti-tumor macrophage function. For example, peripheral blood NK cells from prostate cancer patients exhibit an exhausted phenotype characterized by a decrease of NKG2D and an increase of PD-1 and TIM-3 expression [157]. Of note, PD-1 and TIM-3 are two inhibitory surface molecules. The most known inhibitory axis is PD-1 and its two ligands, PD-L1 and PD-L2, which are overexpressed in some cancers, inhibiting NK cells anti-tumor activity [158, 159]. Consistently, NK cells from prostate cancer patients show a greatly diminished degranulation capacity. These NK cells also secrete soluble factors involved in monocyte recruitment and M2-polarization such as CCL2 and IL-10 [157] (Fig. 2).

#### Myeloid-derived suppressor cells

Myeloid-derived suppressor cells (MDSC) are divided into two groups: polymorphonuclear MDSC (PMN-MDSC), derived from the granulocytic lineage, and monocytic MDSC (M-MDSC) that originate from the myeloid lineage. These cells are known to induce an immunosuppressive TME: M-MDSC through Arginase-1, Nitric Oxide, IL-10 and TGF-β and PMN-MDSC through reactive oxygen species (ROS) and reactive nitrogen species (RNS) production [160].

MDSCs can reduce NK cell’s cytotoxicity. MDSCs from patients with hepatocellular carcinoma inhibit autologous NK cell cytotoxicity and IFNγ production through an NKp30 interaction [161] or by their membrane-bound TGF-β1 [162]. MDSCs also inhibit the Fc receptor-dependent function of NK cells by producing NO [163].

**Fig. 3:**
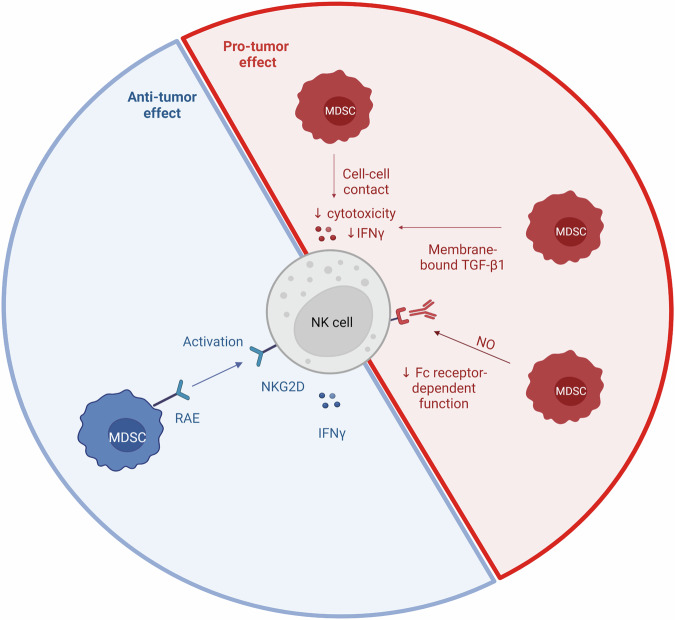
NK cells and MDSC interaction. MDSC are innate immune cells divided into PMN-MDSC and M-MDSC. There are different interactions between MDSC and NK. MDSCs reduce the cytotoxicity, the IFNγ production of NK and their Fc-receptor dependent function. MDSCs also have an anti-tumor effect by expressing Rae, which binds to NKG2D and induces the production of IFNγ by NK cells. Created with BioRender.com.

However, Nausch et al. demonstrated that MDSC can also activate NK cells through their expression of RAE, a ligand of the activating receptor NKG2D. In fact, they showed that NK cells from naive mice co-culture with MDSC produce more IFNγ. This increase was partially reduced using transwell and NKG2D blocking antibodies. Thus, IFNγ production by NK cells was dependent on NKG2D signaling [164] (Fig. 3).

#### Dendritic cells

DCs are innate immune cells that link innate and adaptive immunity through their role as antigen-presenting cells. Through their dendrites, DCs recognize damage-associated molecular patterns (DAMPs), activate and mature. Next, they migrate to T-lymphocyte-rich secondary lymphoid organs, where they present an antigen peptide through the major histocompatibility complex class II (MHC-II). Lymphocytes specific to the antigen peptide will activate, proliferate, and move towards the tumor site. In 1999, an initial study suggested that DCs were capable of initiating NK cell-mediated anti-tumor responses [165]. Subsequent studies showed that DCs and NK cells interact and the last can promote the maturation of DCs, the death of the immature ones and their recruitment to the tumor site. In addition, DCs promote NK cells anti-tumor activity.

NK cells modulate the number of DCs in tumors by producing CCL5 and XCL1, which recruit conventional type 1 dendritic cell (cDC1) [166]. Another study showed an increase in CD103^+^ DCs infiltration in CCL3-secreting CT26 tumor in comparison to WT CT26. CCL3 recruits NK cells, which in turn induced DCs recruitment. Indeed, DCs accumulation was abolished using an anti-Asialo-GM1 antibody, suggesting a role of NK cells in CD103^+^ DCs recruitment [167]. Barry et al. demonstrated that NK cells produce Flt3L, which increase intratumor stimulatory DCs number in mice tumors. Furthermore, the authors found that high levels of NK cells and BDCA3^+^/CD141^+^ DCs (which are intratumoral type I conventional dendritic cells) positively correlate with overall survival and PD-1 blockade outcome in patients suffering from melanoma. Finally, BDCA3^+^ DC/NK cell interaction was also found in humans head and neck squamous cell carcinoma, suggesting that this mechanism could exist as well in other cancer types [168].

Furthermore, NK cells contribute to DC maturation in several way. Firstly, self-specific MHC-I expressing NK cells aid DC maturation in a TNFSF14/LIGHT-dependent manner [169]. Moreover, TNF-α and IFNγ produced in an NKp30-dependent manner by NK cells also contribute to DC maturation [170]. Moreover, coculture with NK cells purified from lung cancer patients containing a subset of CTLA-4^+^ NK cells and DCs from the blood of the same patients results in a decrease of MHC-II and CD86 expression on DCs. This decrease was partially rescued when an anti-CTLA-4 antibody was added to the coculture, suggesting an inhibitory role of CTLA-4-expressing intratumor NK cells [171].

**Fig. 4:**
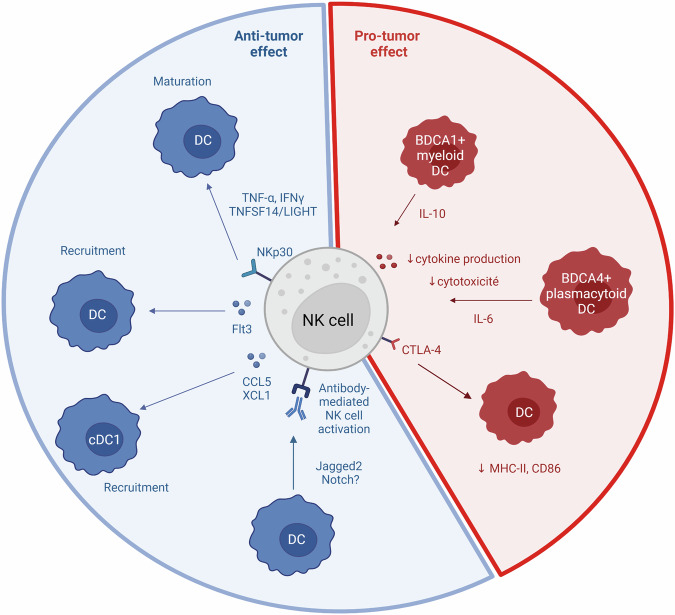
NK cells and DC interaction. DCs are able to increase antibody-mediated NK cell activation but in some other conditions to decrease NK cytokines production and cytotoxicity. In the other part, NK recruits DC and increases their maturation. However, the expression of some immune checkpoints on NK cells is able to decrease DC maturation. Created with BioRender.com.

Finally, DCs can promote antibody-mediated NK cell activation [172] and Jagged2 and Notch could be responsible [173]. Secretion of IL-10 by BDCA1^+^ myeloid DCs and IL-6 by BDCA4^+^ plasmacytoid DCs could in part, be responsible for the inhibition of NK cells cytokine production and cytotoxicity [174] (Fig. 4).

#### Neutrophils

Neutrophils are the most abundant immune cells in the blood: they account for 50-70% of circulating leukocytes in humans. In tumors, they are classified into two phenotypes: an N1 neutrophil phenotype with anti-tumor activity, which induces direct or indirect cytotoxicity by producing TNF-α, CCL3 and expressing ICAM-1. N2 neutrophils are anti-tumor, promote immunosuppression, angiogenesis and produce various cytokines: VEGF, MMP9 CXCL1,2,8,16 and CCL2,3,4,8,12 and 17 [175].

Using the MCA205-Luc mice model, researchers demonstrated that NK cells control tumor growth. Interestingly, in the absence of NK cells, neutrophils over-express VEGF-A and promote angiogenesis. In this context, NK cells seem to limit the pro-tumor effects of neutrophils [176].

**Fig. 5:**
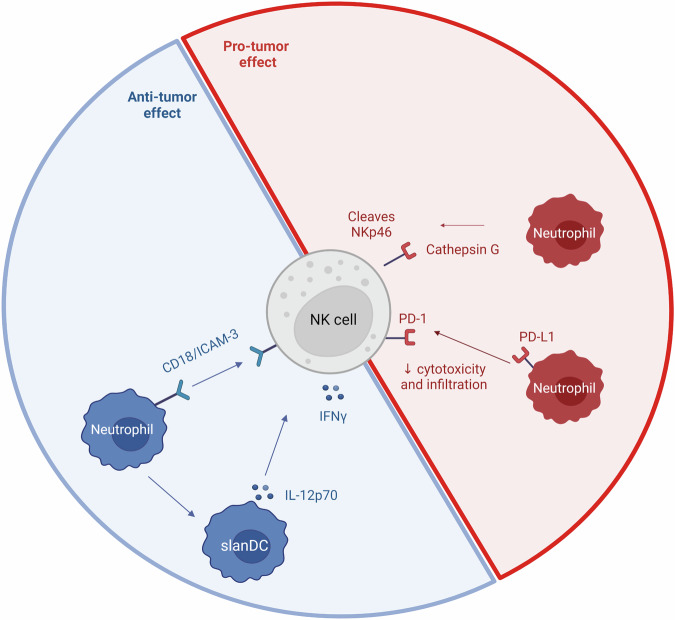
NK cells and neutrophils interaction. Neutrophils are able to increase NK cell IFNγ production directly or indirectly through slanDCs IL-12p70 production. On the other hand, neutrophils can decrease NK cells cytotoxicity and recruitment in tumor. Created with BioRender.com.

Neutrophils can increase NK cell activation. In humans, neutrophils increase IFNγ production by NK cells indirectly by increasing IL-12p70 release by 6-sulfo LacNAc^+^ DCs (slanDCs), a major DCs subset found in the blood and tissues [177] and directly *via* CD18 and ICAM-3 interaction [178]. Neutrophils also show a negative effect on NK cells as neutrophils can inhibit NK cell functions through several mechanisms. First, PD-L1 expressing neutrophils can reduce PD-1^+^ NK cells cytotoxicity and infiltration into tumors [179]. Moreover, neutrophils also produce cathepsin G which cleaves NKp46 on the NK cell’s surface, diminishing their cytotoxic function [180] (Fig. 5).

### NK cells cooperation with adaptative immune cells

#### Mucosal-associated invariant T cells

Mucosal-associated invariant T cells (MAITs) are unconventional T lymphocytes possessing an invariant TCR that exclusively recognizes the MR1 protein presented by MCH class I (MHC-I) [181]. Activation of MAITs occurs either dependently or independently of TCR recognition and results in cytokines and cytotoxic effectors secretion.

A recent study showed that MAITs play a role in regulating the anti-tumor effect mediated by NK cells. In fact, it initially demonstrated that the absence of MAIT strengthens the antitumor effect of NK cells. However, this role seems controversial because it also showed that activated MAIT cells increased NK cell’s anti-tumor functions through IFNγ secretion [182].

#### CD4^+^ T cells

CD4^+^ T-lymphocytes (CD4^+^ T cells) are adaptive immune cells that recognize antigenic peptides associated with MHC-II. Classically, MHC-II expressing cells are antigen-presenting cells: DCs, macrophages and B cells. When T cells are activated, they proliferate and, depending on the cytokine environment, differentiate into a subpopulation: Th1, Th2, Th17, regulatory T cells (Treg), follicular helper T cells (Tfh). These subpopulations will orchestrate the immune response by producing cytokines [183].

Mouse NK cells modulate MHC-II-dependent CD4^+^ T cell responses because NK cells can acquire MHC-II by trogocytosis during direct contact with DCs in vivo. NK cells co-cultured with DCs also expressed low levels of CD80 and CD86, but not enough to be functional [184]. Hence, these NK cells suppress DCs-induced CD4^+^ T cells responses by competing with antigen presentation [184]. The interaction between DCs and NK cells also replaces the help of CD4^+^ T cells in the priming of cytotoxic T lymphocytes [185]. NK cells may also interfere with Tregs, which are immunosuppressive T cells expressing CD4^+^ FOXP3^+^ CD25^+^. In fact, differentiation of Tregs can be prevented by activated NK cells, which interfere with CD28 signaling in CD4^+^ CD25^-^ T cells and inhibit Foxp3 transcription [186]. NK cell depletion also impairs Th1 polarization [187].

In another study, NK cells and CD4^+^ T cells were found to work together to protect mice from melanoma tumor formation in the brain [188]. T cells and notably CD4^+^ T cells help maintain NK cell viability and improve rituximab-mediated NK cells ADCC [189].

**Fig. 6:**
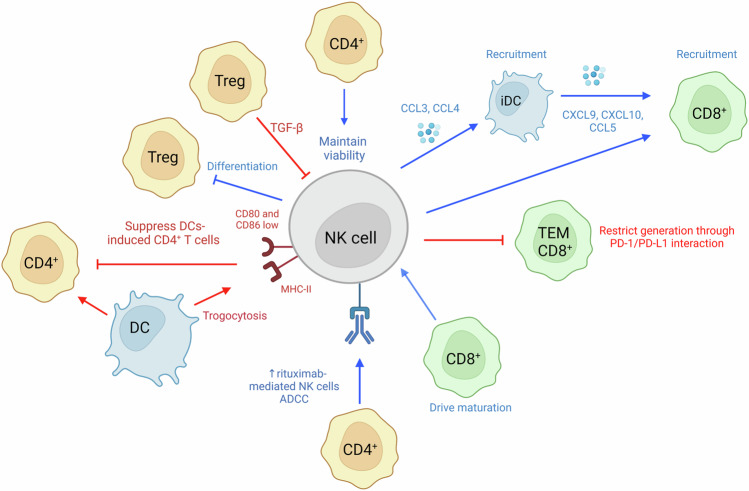
NK cells and T cells interaction. NK are able to suppress DCs-induced CD4^+^ T cells response, to block Treg differentiation, to promote CD8^+^ T cells recruitment directly or indirectly via iDC interaction and to restrict TEM CD8^+^ T cells generation. On their side, adaptive immune cells are able to maintain NK viability, to drive NK maturation, and improve NK cells ADCC. Created with BioRender.com.

In 1999 [190], 2003 [191] and 2004 [192] three different studies showed that Tregs could inhibit NK cell’s functions. The mechanism of interaction was deciphered in 2005 and showed that Tregs inhibit NK cell’s lytic and secretory functions in a TGF-β-dependent manner [193] (Fig. 6).

#### CD8^+^ T cells

NK cells can impact the CD8^+^ T cells response by interacting with DCs, as described above. Moreover, in certain conditions, IL-18-primed NK cells attract iDCs by producing chemokines such as CCL3 and CCL4. In co-culture with iDC, NK cells induce the production of T cells recruiting chemokines such as CXCL9, CXCL10 and CCL5 by iDC [194]. However, NK cells can also restrict TEM CD8^+^ T cell generation indirectly through PD-1/PD-L1 interaction with DC [195].

CD8 + T cells can also drive NK cells maturation. Indeed, in a mice model devoid of CD8 + T cells, liver-resident NK cells appear to be immature, and this can be reverted by CD8 + T cells replenishment. Liver-resident NK cell maturation by CD8 + T cells depends on the CD27/CD70 axis [196].

NK cells can shape the anti-tumor immune response by recruiting T cells through cytokines production. As early as 1994, it was demonstrated that NK cells play a role in T cell recruitment [197]. Indeed, in response to antibody-coated tumor cells, NK cells produce IL-8, MDC, MIP-1 and CCL2, which are chemokines involved in T cells recruitment [198]. Recently, NK cells were found to induce CD8 + T cells recruitment to metastatic site in the B16F10 lung metastasis melanoma model, although the underlying mechanism was not deciphered [28]. Finally, T cells have been also identified to help the anti-tumor immune response mediated by NK cells [199] (Fig. 6).

## Conclusion

Since the first report of “naturally occurring killer lymphocytes”, NK cells have been extensively studied by the scientific community. Their innate ability of recognizing abnormal cells, and their broad cytotoxic arsenal make them an important protagonist in the fight against tumor. Studying how, when and why NK cells target and destroy tumor cells is a key in designing innovative drug to improve NK cells activity. In the tumor bed, NK cells interact with other immune cells through cell-cell contact or cytokines secretion, inducing beneficial or harmful response. Deciphering these interactions could bring new treatment options by targeting new activating or inhibiting pathways to maintain or refuel NK cells activity.

NK cells are gaining more and more attention with the rise of NK-cell-based therapy, CAR-modified or not. Indeed, their safety profile in clinic and the possibility to use them in heterologous settings are two major advantages compared to CAR-T cell therapy. However, their clinical efficacy in monotherapy is deceiving and there is a need to develop new cotreatments [200]. The knowledge summarized here could help to improve NK cells’ cytotoxic activity as well as predict how ex vivo expanded NK cells infused in patients will behave in the tumor bed.

## Data Availability

The relevant information is available from the corresponding authors upon reasonable request.
